# A New Insight on Carcass and Meat Quality in Ruminants

**DOI:** 10.3390/ani12223153

**Published:** 2022-11-15

**Authors:** Guillermo Ripoll, Begoña Panea

**Affiliations:** 1 Animal Science Department, Centro de Investigación y Tecnología Agroalimentaria de Aragón (CITA), Avda. Montañana 930, 50059 Zaragoza, Spain; 2 Agrifood Institute of Aragon-IA2 (CITA-Zaragoza University), Avda. Miguel Servet 177, 50013 Zaragoza, Spain

## 1. Introduction

Ruminant production systems are very important in many areas of the world and a key aspect of the economy and culture. Ruminants play an important role in low-income areas because they can graze marginal pastures that other species cannot use, or even are agricultural high natural value systems [1]. Moreover, these systems fix the population to unfavorable rural areas and even can prevent forest fires. Although meat consumption is increasing worldwide [2], the production of meat from ruminants is decreasing together with the number of farms, especially in Europe [3]. Production systems are important because they directly affect carcass and meat composition and quality. The interactions between diet, the animal’s physiological status, and the environment will impact the yield, composition, quality, and sensorial appeal of its meat products; so, the use of forages, novel feeds, industry by-products, and more sustainable production systems for holistic benefit could modify the value of meat products delivered for human consumption and this must be considered [4].

Food quality is a complex term that includes, in addition to safety, such intrinsic characteristics as appearance, color, texture, and flavor, which are modified by both pre- and post mortem factors. Meat has a short shelf life, and new insights into methods of preservation are gaining interest in preventing deterioration and ensuring the absence of foodborne microorganisms and pathogenic bacteria which lead to meat spoilage. These reactions lead to a loss of the nutritional and sensory qualities of the meat products. For this Special Issue, we are also interested in studies on any of these factors or preservation methods for improving the quality and shelf-life of meat. A quick search with the key words shows around 2500 papers from 2020 to now dealing with this topic. This reveals that carcass and meat quality in ruminants is still a hot topic. Novel strategies can be implemented in production, preparation, storage, and distribution systems to induce qualitative and quantitative changes in meat product composition and to optimize the beneficial properties for human health [5]. On the other hand, meat and meat products contain essential components of the human diet such as protein of high biological value, essential fatty acids, vitamins with high bioavailability, etc. Meat products are nowadays perceived as less healthy and less attractive by consumers, and this makes them more selective in the products they consume, as they are increasingly aware of improving their health through the foods they consume [6]. There is a new trend in the production of healthier meat products to satisfy consumer demands.

This Special Issue of *Animals*, entitled “Carcass and Meat Quality in Ruminants”, aims to compile the recent literature with a focus on carcass development, quality, and valorization in addition to meat quality. It includes ten original research articles about various types of meat from ruminant species (bovine [7,8,9,10,11], ovine [12,13,14], and caprine [15,16]) as well as one review article about strategies to reduce *E. Coli* contamination [17]. These articles, while their aims are different, provide a deep insight on the current topics of carcass and meat science of ruminants.

## 2. Summary of Published Papers

Indigenous veld goats (IVG) are a group of specific pure-breed indigenous eco-types represented by the IVG Association, which defines specific standards that a goat must adhere to. These eco-types are characterized by large frames and competitive meat yield, them being animals with disease resistance and adaptability to harsh climates. The paper of van Wyk, Hoffman, Strydom, and Frylinck [15] focuses on the effect of breed and castration on the meat quality of six different muscles to establish quality baselines for IVG eco-types. Various meat quality characteristics of six muscles from large-frame Boer goats and indigenous veld goats were studied. The animals were raised on hay and natural grass, and on a commercial pelleted diet to a live weight of 30–35 kg. All goats were slaughtered at a commercial abattoir and the dressed carcasses were chilled at 4 °C within 1 h post mortem. The muscles were dissected from both sides 24 h post mortem and aged for 1 d and 4 d. Variations in meat characteristics such as ultimate pH, water-holding capacity, drip loss, myofibril fragment length, intramuscular fat, connective tissue characteristics, and Warner–Bratzler shear force were recorded across muscles. Bucks had higher lightness and hue-angle values, whereas wethers had increased redness and chroma values. This study alleviates some misconceptions that exist about the potential quality of “indigenous” goat meat. More muscle meat quality differences were found between sexes than between breeds, while large-frame IVGs consisted of a mixture of the different goat eco-types. In addition, the study further showed that goat muscles have different characteristics from those of other red-meat animals and the muscle baseline data will allow informed decisions to support muscle-specific marketing strategies, which may be used to improve consumer acceptability of chevon.

The aim of the study by Sánchez, Marti, Verdú, González, Font-i-Furnols, and Devant [11] was to characterize three different commercial beef-fattening systems. The fattening systems were intensive Mediterranean fattening programs with different sex, breed, nutrition, and days on feed. Fattening systems were described according to their performance, behavior, and carcass and meat quality when raised simultaneously under the same housing and care conditions. In the authors’ words, data generated from this study are the first step for decision making and offer technical information to consider whether raising crossbred Angus bulls can be a good alternative to Holstein bulls in a Mediterranean dairy beef-fattening system. Authors did not find great differences in efficiency, intramuscular fat, or meat tenderness among the three Mediterranean production systems evaluated. As a relevant finding, it was reported that the purchase decision was indicative of an unforeseen impairment in meat quality. In summary, according to the present study, a transition from a production system based on Holstein bulls to crossbred Angus is only reasonable to improve carcass conformation, and only marketing approaches for meat distinction could strengthen this decision.

Mohd Azmi, Mat Amin, Ahmad, Mohd Nor, Meng, Zamri Saad, Abu Bakar, Abdullah, Irawan, Jayanegara, and Abu Hassim [9] examined the effect that a mixture of 4% bypass fat and 26% concentrate supplementations in the buffalo basal diet had on both the carcass characteristics and the proximate and fatty acid composition in three muscles of Murrah cross and swamp buffaloes. Additionally, they studied the profitability of raising buffaloes. The results showed that supplemented bypass fat significantly increased the pre-slaughter weight, hot and cold carcass weights, meat-to-fat ratio, pH, moisture, and crude protein, while the carcass yield and carcass fat percentages were significantly decreased. Furthermore, Murrah cross showed a significantly higher pre-slaughter weight, hot and cold carcass weights, carcass bone percentage, and total fatty acid, but a lower meat-to-bone ratio when compared to swamp buffaloes. Supplementing using bypass fat increased the cost of buffalo feeding but resulted in a higher revenue and net profit. In conclusion, the concentrate and bypass fat supplementations in the buffalo diet could alter the nutrient compositions of buffalo meat without a detrimental effect on carcass characteristics, leading to a higher profit.

The study performed by Bharanidharan, Thirugnanasambantham, Ibidhi, Bang, Jang, Baek, Kim, and Moon [8] focused on the influence of dietary protein level on growth performance, fatty acid composition, and the expression of lipid metabolic genes in intramuscular adipose tissues from 18- to 23-month-old Hanwoo steers, representing the switching point of the lean-to-fat ratio. It was observed that the high-protein diet significantly increased the expression of intramuscular *PPARα* and *LPL* while it did not affect to genes involved in fatty acid uptake, such as *CD36* and *FABP4,* nor lipogenesis, such as *ACACA*, *FASN*, and *SCD*. In addition, it downregulated intramuscular *VLCAD* related to lipogenesis but also *GPAT1*, *DGAT2*, and *SNAP23*, which are involved in fatty acid esterification and adipocyte size. Hanwoo steers fed a high-protein diet at 18–23 months of age resulted in a relatively lower lipid turnover rate than steers fed a low-protein diet, which could be responsible for shortening the feeding period. These results showed a low lipid turnover rate, which could be responsible for shortening the feeding period. Furthermore, Hanwoo steers fed a high-protein diet during this period showed increased intramuscular fatty acid content, oleic acid, and fineness in the marbling texture during later life by downregulating *SNAP23*.

Because aromatic plant distillation residues are being considered with growing interest in a two-fold object, enhancing meat quality by increasing the antioxidant properties and reducing feed prices, Yagoubi, Smeti, Ben Said, Srihi, Mekki, Mahouachi, and Atti [12] studied the effects of rosemary distillation residue incorporation in concentrate associated with two nitrogen sources as a substitute for standard concentrate on lambs’ growth, carcass traits, and meat quality. Growth, carcass weights, dressing percentages, and non-carcass component weights were unaffected by the diet. Moreover, regional and tissular compositions and meat physical properties including color were similar irrespective of the diet. However, meat produced by lambs receiving rosemary distillation residue-based concentrate was richer in vitamin E and polyphenol contents than the control lambs. Rosemary by-products may substitute the standard concentrate resulting in similar lamb growth and carcass traits, while improving meat quality by increasing vitamin E content, which could improve its antioxidant power. The results provide evidence that the use of rosemary residues as a cereal substitute up to 30% in concentrate for sheep feeding did not alter animal performances. This smart strategy of using aromatic plant by-products could be effective especially in the Mediterranean region, where this by-product is available in an plentiful amount and is free. The cost per kilogram of meat produced by Barbarine lambs was reduced to 40%. In addition, faba bean (*Vicia Faba*) could be used as a substitute to soybean without affecting carcass nor meat quality; this nitrogen source could potentially be produced, given its production is relatively cheap compared to the nutritional value, to reduce the import of soybean meal, which is still expensive.

Sainfoin is a forage legume with a medium content of proanthocyanidins (PAC), which may affect animal performance and product quality. Therefore, Baila, Lobon, Blanco, Casasus, Ripoll, and Joy [13] studied the effect of PAC from sainfoin fed to dams, using polyethylene glycol as a blocking agent, on the performance and carcass and meat quality of their suckling male lambs. They found that the presence of PAC in the dams’ diet did not affect the growth, blood metabolites, and carcass weight and fatness of the suckling lambs but decreased the lightness of caudal fat and increased the weight of the digestive compartments. Regarding the meat characteristics, PAC only decreased polyphenol content. The inclusion of PAC from sainfoin in the dams’ diet had no significant effect on the ADG, plasmatic antioxidant activity, and carcass and meat quality of their suckling lambs. Therefore, fresh sainfoin can be fed to ewes during lactation to produce suckling lambs, achieving good performance and meat quality.

The use of pea has been recommended to replace soybean meal in the diet of ruminants, but it may affect meat quality. However, the title of the study of Blanco, Ripoll, Lobon, Bertolin, Casasus, and Joy [14] (The Inclusion of Pea in Concentrates Had Minor Effects on the Meat Quality of Light Lambs) speaks for itself. The aim of this study was to evaluate the effect of the proportion of pea (0%, 10%, 20%, and 30%) in fattening concentrates fed to light lambs for 41 days on carcass color and meat quality. Pea inclusion affected neither the color of the lamb carcasses nor affected most of the parameters of the meat quality. However, the inclusion of pea affected the cholesterol content, and the 20% pea concentrate yielded meat with greater cholesterol contents than the 30% pea concentrate did. The inclusion of pea had minor effects on individual FAs but affected the total saturated fatty acids (*p* < 0.01) and the thrombogenicity index. A greater total saturated fatty acid content was recorded for the 20% pea concentrate than for the rest of the concentrates, and a greater thrombogenicity index was recorded for the 20% concentrate than for the 10% pea concentrate. The results indicated the viability of the inclusion of pea in the fattening concentrate of light lambs without impairing meat quality, with the 30% pea concentrate being the most suitable to reduce the soya dependency.

Since goat milk has a higher value than kid meat in Europe, some farmers rear kids with milk replacers, although some studies have stated that kids raised on natural milk yield higher-quality carcasses. With the aim of enlightening this topic, Ripoll, Alcalde, Argüello, Córdoba, and Panea [16] evaluated the influence of the use of milk replacers on several carcass characteristics of suckling kids from eight Spanish goat breeds. For all studied variables, interactions were found between the rearing system and the breed. In general, the milk replacer increased the head and visceral weights, as well as the length measurements and muscle percentages. Conversely, the natural milk-rearing system increased carcass compactness and resulted in higher fat contents, independent of the deposit. The choice of one or another rearing system should be made according to the needs of the target market.

The amount and distribution of subcutaneous fat are important factors affecting beef carcass quality. The degree of fatness is determined by visual assessments scored on the SEUROP system. New technologies such as the image analysis method have been developed and applied to enhance the accuracy and objectivity of this classification system. In the study by Mendizabal, Ripoll, Urrutia, Insausti, Soret, and Arana [7], 50 young bulls were slaughtered and after slaughter, the carcasses were weighed and a SEUROP system fatness score assigned. A digital picture of the outer surface of the left side of the carcass was taken and the area of fat cover (fat area) was measured using an image analysis system. Commercial cutting of the carcasses was performed 24 h post mortem. The fat trimmed away on cutting (cutting fat) was weighed. A regression analysis was carried out for the carcass cutting fat on the carcass fat area to establish the accuracy of the image analysis system. A greater accuracy was obtained by the image analysis (R^2^ = 0.72; *p* < 0.001) than from the visual fatness scores (R^2^ = 0.66; *p* < 0.001). The findings of this study suggest that measuring carcass fat area using an image analysis can be regarded as a suitable indicator of carcass fatness in young bulls of Spanish meat breeds. Furthermore, including this assessment method in the framework of the EU’s SEUROP classification system could be worthwhile because it provides an objective measure of carcass fatness. Nevertheless, before applying an image analysis to other breeds or production systems, the method should be tested on the carcasses of fatter animals spanning the broadest possible range of fatness scores and, if it is feasible, spanning the entire interval from 1 to 5.

In autochthonous dairy cattle farms, the production of salami could represent an alternative commercial opportunity. However, the diet of animals can modify the quality of meat and meat products. Therefore, a study was carried out by Alabiso, Maniaci, Giosue, Di Grigoli, and Bonanno [10] to investigate the fatty acid composition of salami made using the meat from grazing or housed young bulls and grazing adult cows of the Cinisara breed. Animal category influenced the FA composition, although the addition of lard mitigated the differences found in fresh meat. The salami from grazing animals showed higher polyunsaturated fatty acid content and a higher level of linoleic acid than that from other animal categories. Salami made from grazing adult cows’ meat showed a lower polyunsaturated/saturated fatty acid ratio, but a better *n-6*/*n-3* ratio compared to housed bulls due to the lower content of linoleic acid. Multivariate analysis showed an important influence of animal category on fatty acid composition due to age, feeding system, and meat fat content of animals, despite the addition of lard.

Finally, closing the Special Issue is a review entitled “Preharvest Management and Postharvest Intervention Strategies to Reduce Escherichia coli Contamination in Goat Meat: A Review by Kannan, Mahapatra and Degala [17]. While researchers have long focused on postharvest intervention strategies to control *E. coli* outbreaks, recent works have also included preharvest methodologies. In goats, these include minimizing animal stress, manipulating the diet a few weeks prior to processing, feeding diets high in tannins, controlling feed deprivation times while preparing for processing, and spray washing goats prior to slaughter. The postharvest intervention methods studied in small ruminant meats have included spray washing using water, organic acids, ozonated water, and electrolyzed water, and the use of ultraviolet (UV) light, pulsed UV-light, sonication, low-voltage electricity, organic oils, and hurdle technologies. These methods show strong antimicrobial activity and are considered environmentally friendly. However, cost-effectiveness, ease of application, and possible negative effects on meat quality characteristics must be carefully considered before adopting any intervention strategy for a given meat-processing operation. As discussed in this review paper, novel pre- and postharvest intervention methods show significant potential for future applications in goat farms and processing plants.

## 3. Conclusions

This Special Issue on the theme of carcass and meat quality in ruminants has attracted the interest of authors from all over the world, publishing one review and ten original research papers.

Progress has been made in the topic investigated, but it is still necessary to increase the research on it due to the extensiveness of this field and the peculiarities and special problems of the different species of ruminants.

Finally, I would like to conclude that the manuscripts found in this Special Issue have been submitted by internationally recognized research teams. I would like to thank all authors for their contributions.
